# Extended septal myectomy versus alcohol septal ablation: clinical results at a national referral centre

**DOI:** 10.1093/icvts/ivae058

**Published:** 2024-04-03

**Authors:** Juan Esteban de Villarreal-Soto, Juan Francisco Oteo-Domínguez, Daniel Martínez-López, Elsa Carolina Ríos-Rosado, Beatriz Vera-Puente, Jean Carlo Olivo-Soto, Fernando Arízaga-Arce, Pablo García-Pavía, Víctor Manuel Ospina Mosquera, Susana Villar García, Jessica García Suárez, Miguel Ángel Cavero, Carlos Esteban Martín-López, Alberto Forteza-Gil

**Affiliations:** Cardiac Surgery, Puerta de Hierro Majadahonda University Hospital, Majadahonda, Spain; Cardiology Department, Puerta de Hierro Majadahonda University Hospital, Majadahonda, Spain; Cardiac Surgery, Puerta de Hierro Majadahonda University Hospital, Majadahonda, Spain; Cardiac Surgery, Puerta de Hierro Majadahonda University Hospital, Majadahonda, Spain; Cardiac Surgery, Puerta de Hierro Majadahonda University Hospital, Majadahonda, Spain; Cardiac Surgery, Puerta de Hierro Majadahonda University Hospital, Majadahonda, Spain; Cardiac Surgery, Puerta de Hierro Majadahonda University Hospital, Majadahonda, Spain; Cardiology Department, Puerta de Hierro Majadahonda University Hospital, Majadahonda, Spain; Cardiac Surgery, Puerta de Hierro Majadahonda University Hospital, Majadahonda, Spain; Cardiac Surgery, Puerta de Hierro Majadahonda University Hospital, Majadahonda, Spain; Anesthesia Department, Puerta de Hierro Majadahonda University Hospital, Majadahonda, Spain; Cardiology Department, Puerta de Hierro Majadahonda University Hospital, Majadahonda, Spain; Cardiac Surgery, Puerta de Hierro Majadahonda University Hospital, Majadahonda, Spain; Cardiac Surgery, Puerta de Hierro Majadahonda University Hospital, Majadahonda, Spain

**Keywords:** Hypertrophic obstructive cardiomyopathy, Septal myectomy, Alcohol septal ablation, Inverse-probability weighted regression-adjustment

## Abstract

**OBJECTIVES:**

Extended septal myectomy and alcohol septal ablation are 2 invasive treatments for hypertrophic obstructive cardiomyopathy. Our goal was to compare which of these techniques achieved a higher reduction in gradients, improvement in New York Heart Association (NYHA) functional class and reduction in medical treatment.

**METHODS:**

It is a single-centre observational and retrospective analysis. We used multivariable regression analyses to assess the association of ablation/myectomy with different outcomes. The odds ratio or coefficient along with the 95% confidence interval was estimated according to the group and adjusted for the corresponding preprocedural variables and EuroSCORE II.

**RESULTS:**

A total of 78 patients underwent septal myectomy, and 25 patients underwent alcohol septal ablation. Basal and Valsalva gradients after myectomy were reduced to a higher degree in comparison to ablation: 21.0 mmHg [*P* < 0.001, 95% confidence interval -30.7; -11.3], and 34.3 mmHg (*P* < 0.001, -49.1; -19.5) respectively. Those patients who received a myectomy had a lower probability of having moderate mitral regurgitation (odds ratio = 0.18, *P* = 0.054). Patients after septal myectomy were more likely to be NYHA functional class I (80.4%), whereas patients after ablation were more likely to be NYHA functional class III (48%). Both groups continued with beta-blocker therapy, but disopyramide could be discontinued after the myectomy in more cases (20%–36% vs 59%–1.3%; *P* < 0.001), and there was a tendency to discontinue calcium channel blockers (48%–16% vs 15.4–3.8%; *P* = 0.054).

**CONCLUSIONS:**

After adjustment using preprocedural gradients and EuroSCORE II, myectomy achieves greater reduction in left ventricular outflow tract gradients compared to septal ablation.

## Graphical Abstract

**Figure:**
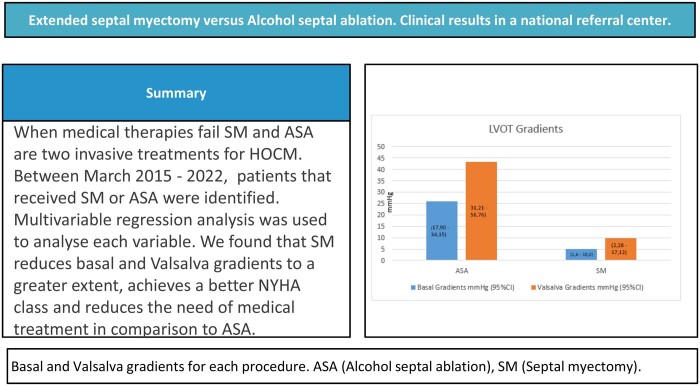

## INTRODUCTION

Hypertrophic obstructive cardiomyopathy (HOCM) is a genetic and familiar disease with an incidence that oscillates between 0.2% and 0.5% [1–2]. The main prognostic factor for HOCM is left ventricle outflow tract (LVOT) obstruction. Thus treatments are targeted to relieve this condition [3]. The systolic anterior motion (SAM) of the anterior leaflet of the mitral valve contributes to the obstruction, but there are also other anomalies that have been related to LVOT obstruction such as anomalies in the papillary muscles or the elongation of the anterior leaflet of the mitral valve. Medical treatment with the beta blocker disopyramide and the calcium channel blockers (CCB) are used to relieve symptoms. New therapies such as Mavacamten have shown benefits in some groups of patients [4]. However, in patients with persistent symptoms despite medical therapy, invasive treatment is needed.

Septal myectomy (SM) was first developed in 1958 in Cleveland, OH, USA [5]. The technique underwent many changes [6] until it was perfectioned at the Mayo clinic in the 1980s with the extended myectomy, as we know it today [7]. Since then, extended SM has been considered the gold standard for HOCM. In large series, this technique has a surgical risk of less than 1%, and long-term post-myectomy survival is similar to that of the general population [8]. On the other hand, alcohol septal ablation (ASA) was introduced in 1994 as a less invasive solution compared with SM [5]. However, complications such as atrioventricular block [6] and increased gradients in the LVOT despite the procedure with the need of reintervention [7] have been reported.

There is no evidence in the literature of randomized trials comparing ASA versus SM. The small number of patients affected by this condition would not permit us to have enough cases in each arm to extract solid conclusions [11–13].

## MATERIALS AND METHODS

We conducted an observational retrospective study based on a single-centre database. Patients are included in the database prospectively. Patients aged 18 or older in whom an SM or ASA was performed from March 2015 to March 2022 were included in the analysis. The decision to perform an SM or an ASA was made by the heart team. Patients who underwent apical myectomy or mitral valve replacement were excluded from the analysis.

Electronic medical records were collected and reviewed to identify baseline characteristics and procedural information. Post-procedure images were reviewed meticulously before including them in the database. Follow-up information was retrieved from the electronic medical record of each patient in the outpatient clinic, and patients were contacted by telephone if they were being reviewed somewhere else.

### Ethical statement

The study complied with the provisions in European Union and Spanish legislation on data protection and the Declaration of Helsinki 2013. Our institutional ethics committee waived the need for ethics approval and informed consent for the collection, analysis and publication of this non-interventional study.

#### Alcohol septal ablation technique

Coronary arteriography is required to exclude coronary artery disease and to define a potential target septal artery; the first large septal branch or one of the first side branches is considered the target artery. A temporary pacemaker lead is inserted in patients without a permanent pacemaker or implantable cardioverter defibrillator.

A guidewire is advanced into the target septal artery; an over-the-wire balloon is then advanced into the target septal artery and inflated to avoid backflow of alcohol into the left anterior descending artery, thereby avoiding infarction of non-target myocardial areas. Echocardiographic contrast agent is injected through the balloon catheter with simultaneous transthoracic echocardiography. Selective angiography of the target septal branch through the inflated balloon catheter should document the adequate sealing of the septal branch and exclude filling of any other coronary artery through septal collaterals. Then, 1.5–2 ml of absolute alcohol is injected slowly through the central lumen of the balloon catheter under continuous fluoroscopic, haemodynamic and electrocardiographic observation. The quantity of injected alcohol is determined by the septal thickness or septal artery diameter. Alcohol is injected for 3 to 5 min, followed by a normal saline flush (1 ml for 2 min). Balloon occlusion is maintained for at least 10 min. Additional septal arteries may be treated if there is significant residual LVOT obstruction and if other septal arteries are accessible. After withdrawal of the balloon catheter, a final angiogram is performed to document complete occlusion of the septal branch and normal flow in the left anterior descending artery. Provocative gradients are measured with the Valsalva manoeuvre after the procedure if basal gradients are normal with high Valsalva gradients preprocedure. At least 36–48 h of haemodynamic and electrocardiographic surveillance are necessary.

#### Septal myectomy technique

Prior to beginning cardiopulmonary bypass (CPB), intraoperative transoesophageal echocardiography (TEE) is performed to reassess the distance between the greatest septal thickening and the aortic annulus, the anterior leaflet length, papillary muscle disposition and LVOT gradients. The LVOT gradients are also measured directly before CPB. Two guidewires are inserted, one in the ascending aorta and the other in the left ventricle through the right ventricle.

After initiating CPB, aortic clamping and cardioplegia infusion, an oblique or hockey stick aortotomy is performed; it is extended through the noncoronary sinus up to 1 cm above the aortic annulus. A number 10 scalpel blade is used to initiate the resection. The incision is started from the midpoint of the right sinus, 1 cm from the insertion of the aortic leaflet and in a counterclockwise direction from the surgeon's position. The muscle adjacent to the right fibrous trigone is not resected to prevent atrioventricular block. The thickness of the scalpel blade is used as a reference for depth, so by deepening the entire blade we achieve resections of 1 cm. After resection of this first segment of myocardium, the assistant applies pressure to the right ventricle with a swab supported by Foerster forceps to improve the exposure, and the resection is extended to a midventricular level. A resection of 3 to 12 grams of cardiac muscle tissue is considered adequate. Anomalous chordae tendineae originating from the mitral leaflet and inserting directly into the ventricular free wall or septum should be resected. Also, a vertical plication in those with an elongated anterior mitral valve leaflet with a length >34 mm is performed [26]. After a careful review of the septum and removal of possible remains of muscle, we close the aortotomy and exit CPB. Verification of the result of myectomy is performed by TEE and by measuring direct gradients.

#### Measurement of provocative direct gradients after a septal myectomy

Although the absence of a significant gradient has previously been demonstrated with TEE, it is also recommended to perform direct measurements of the gradients in the aorta and left ventricle to verify the absence of residual obstruction. Provocative gradients of less than 10 mmHg are acceptable. We used 2 provocative manoeuvres: provocation of a premature ventricular contraction (Brockenbrough-Braunwald-Morrow manoeuvre) and dobutamine at 5 µcg/kg/min, increasing its dose progressively until 20 µcg/kg/min, simulating the Valsalva manoeuvre. If gradients with provocative manoeuvres are greater than 15 mmHg, myectomy is considered suboptimal. Therefore new clamping and extended myectomy are performed.

### Primary and secondary outcomes

Our primary outcome was to analyse the decrease in the gradients at the LVOT level with the SM versus the ASA procedure. Secondary outcomes were to evaluate the improvement in New York Heart Association (NYHA) functional class and the need for medical treatment after discharge. We also analysed the complications associated with each procedure, the requirement for a subsequent invasive procedure due to symptoms and/or residual gradient, the hospital length of stay (LOS) and the in-hospital and follow-up deaths. A complication is defined as any problem that may arise during or after the operation that was not an intentional effect of the operation and that may cause any harm to the patient. The possible complications that we examined were ventricular septal defect, stroke, postoperative bleeding, atrioventricular block, atrial fibrillation, infective endocarditis and the requirement for mitral valve replacement.

### Statistical analyses

Descriptive analyses were performed by means of absolute and relative frequencies for categorical variables and median and 25th and 75th percentiles for numerical variables.

We have assessed the association of the procedure with the different outcomes through multivariable regression analyses. Linear regression analyses were applied whenever the dependent variable was numerical, such as basal and Valsalva gradients, left atrium volume and index left atrial volume, left ventricle end-systolic volume and end-diastolic volume. The coefficient along with the corresponding 95% CI was estimated. A logistic regression model was applied for dichotomous dependent variables–SAM of the anterior leaflet of the mitral valve and mitral regurgitation–and it showed the odds ratio (OR) with the corresponding 95% CI. In every model, we made adjustments using the corresponding preprocedural variable and EuroSCORE II.

Because the EuroSCORE II predicts hospital mortality and considers many clinical variables, we used this variable to adjust the multivariable models. The EuroSCORE II model considers 18 variables (age, sex, extracardiac arterial disease, chronic lung disease, renal function, poor mobility, previous cardiac surgery, active infective endocarditis, critical preoperative status, insulin-dependent diabetes, angina at rest, left ventricular ejection fraction, myocardial infarction within 90 days, pulmonary hypertension, level of urgency of surgery, weight of intervention and surgery of the thoracic aorta) [12].

The post-procedural NYHA functional class was measured at different times for all patients, but we know that after the third month after the procedure, the functional class is stable with time. Therefore we restricted the analyses of NYHA functional class to those patients who were examined at least 3 months after the procedure.

Overall survival curves are shown and were estimated using the Kaplan–Meier method. The level of significance was set at 0.05. Stata v17 software (StataCorp LP, College Station, TX, USA) was used in the statistical analysis.

## RESULTS

A total of 78 patients underwent SM (mean age, 58 years old; 45% females), and 25 patients underwent ASA (mean age, 70 years old; 68% females). Patient baseline characteristics are shown in Table 1. The cardiopulmonary bypass time was 58.23 (30.89) min, and the aortic cross-clamp time was 43.61 (24.89) min. Table 2 summarizes post-procedural results with the main difference found in the basal and Valsalva gradients. In the SM group, a median of 0 versus 18 mmHg in the ASA group for basal LVOT gradients was found (*P* < 0.001), and a median of 0 was found in the SM group versus 35 mmHg in the ASA group for the Valsalva LVOT gradients (*P* < 0.001). No reintervention due to bleeding was required in any of the groups. One patient presented with a transient neurologic deficit in the SM group (0% vs 1.28%; *P* = 0.868). Reintervention due to cardiac tamponade postoperatively occurred in 2 patients in the SM group (0% vs 2.78%; *P* = 0.811). One patient in the SM group presented with a ventricular septal defect that was observed intraoperatively during TEE (0% vs 1.28%; *P* = 0.868). The ASA group had a higher rate of pacemaker implants (16% vs 2.6%; *P* = 0.013); 1 patient in the ASA group needed a pacemaker before the procedure, so the procedure was carried out in the cardiac catheterization laboratory. Days in the intensive care unit (ICU) and the LOS in the hospital in the ASA group were 2 and 6 days, respectively. Meanwhile in the SM group, the median of the ICU and hospitalization days was 3 and 8 days, respectively (ASA vs SM ICU stay, *P* = 0.031; ASA vs SM hospitalization stay, *P* < 0.001).

**Table 1:** Baseline characteristics, medical treatment and echocardiography data

Baseline characteristics		ASA	SM	*P-*value
Age		70.4 (9.12)	57.82 (11.81)	<0.001
Female		17 (68%)	35 (44.9%)	0.044
Weight (kg)		76.13 (13.76)	80.23 (13.95)	0.210
BMI (kg/m2)		29.19 (5.63)	29.70 (4.71)	0.663
BSA (m2—Mosteller)		2.63 (3.78)	1.93 (0.18)	0.102
EuroSCORE II		2.09 (1.53)	2.1 (2.25)	0.282
NYHA	I	0 (0%)	1 (1.3%)	0.407
	II	5 (20%)	10 (13.5%)	
	III	20 (80%)	57 (77%)	
	IV	0 (0%)	6 (8.1%)	
ECG sinus rhythm		18 (72%)	58 (78.4%)	0.647
	Atrial fibrillation	5 (20%)	11 (14.9%)	
Pacemaker		4 (16%)	2 (2.7%)	0.018
Implantable defibrillator		0 (0%)	16 (21.9%)	0.004
Dyspnoea		25 (100%)	78 (100%)	0.562
Angina		19 (76%)	23 (29.4%)	<0.001
Syncope		1 (4%)	10 (12.8)	0.191
Beta blockers		25 (100%)	74 (94.9%)	0.086
Calcium channel blockers		12 (48%)	12 (15.4%)	0.001
Disopyramide		5 (20%)	46 (59%)	<0.001
Septal thickness (mm), mean (SD)		19.56(3.22)	21.95(3.83)	0.057
SAM		21 (84%)	61 (78.2%)	0.815
Mitral regurgitation ≥3		8 (32%)	48 (61.6%)	0.008
Basal LVOT gradients (mmHg), median (p10; p90)		68.5 (13; 124)	70 (21.5; 129.5)	0.958
Valsalva LVOT gradients (mmHg), median (p10; p90)		93 (57; 164)	104 (51; 170)	0.432
Previous procedure		2 (8%)	2 (2.7%)	0.354

Categorical variables are shown as frequency and percentage. Quantitative variables are shown as mean and standard deviation.

ASA: alcohol septal ablation; BMI: body mass index; BSA: body surface area; ECG: electrocardiogram; LVOT: left ventricle outflow tract; NYHA: New York Heart Association; SAM: systolic anterior motion; SD: standard deviation.

**Table 2:** Post-procedural results

Post-procedural results		ASA	SM	*P-*value
NYHA	I	3 (12.5%)	56 (81.16%)	<0.001
	II	10 (41,67%)	12 (17.39%)	
	III	11 (45,83%)	1 (1.45%)	
	IV	0 (0%)	0 (0%)	
ECG sinus rhythm		18 (72%)	58 (78.4%)	0.004
	Atrial fibrillation	5 (20%)	11 (14.9%)	
New pacemaker implantation		4 (16%)	2 (2.6%)	0.013
Dyspnoea		12 (75%)	8 (10.3%)	<0.001
Angina		2 (12.5%)	2 (2.6%)	0.115
Syncope		0 (0%)	0 (0%)	—
Beta blockers		24 (96%)	56 (71.8%)	0.034
Calcium channel blockers		4 (16%)	3 (3.8%)	0.054
Disopyramide		9 (36%)	1 (1.3%)	<0.001
Septal thickness (mm), mean (SD)		17.94(3.98)	15.31(3.82)	0.028
SAM		9 (36%)	17 (21.8%)	0.482
Mitral regurgitation ≥3		4 (16%)	4 (5.1%)	0.323
Basal LVOT gradients (mmHg), median (p10; p90)		18 (0; 70)	0 (0; 17)	<0.001
Valsalva LVOT gradients (mmHg), median (p10; p90)		34.5 (8; 90)	0 (0; 53)	<0.001

Categorical variables are shown as frequency and percentage. Quantitative variables are shown as mean and standard deviation.

BMI: body mass index; BSA: body surface area; ECG: electrocardiogram; LVOT: left ventricle outflow tract; NYHA: New York Heart Association Class; SAM: systolic anterior motion; SD: standard deviation.

The SM group had an average of 21.0 mmHg less of an LVOT basal gradient than those in the ASA group [*P* < 0.001; 95% confidence interval (CI), -30.72; -11.33]. On average, the Valsalva LVOT gradient after SM was 34.29 mmHg less than that of the ASA group (*P* < 0.001; 95% CI -49.10; -19.47). We performed a second cross-clamping in 16 patients (20%) due to provocative gradients >15 mmHg.

Those patients who received SM had a lower proportion of moderate mitral regurgitation (MR) (OR= 0.18, *P* = 0.054), though the differences did not reach statistical significance. Predictive probability for MR in the ASA group revealed a 21% risk (95% CI 2.7; 39.8) of developing moderate MR, whereas SM had a 5% risk (95% CI, -0.01; 0.10). Patients who underwent myectomy were half as likely to exhibit SAM as those undergoing ablation, without being statistically significant (*P* = 0.21). The OR of those receiving SM was 0.51 (95% CI, 0.18; 1.47). In the SM group, mitral valve repair with vertical plication was associated in 17 patients (21.79%) and resection of anomalous chordae tendineae in 13 patients (16.67%).

Because the NYHA functional class can be affected in the earlier post-procedural days and is virtually unchanged after 3 months after the procedure, we have eliminated those patients with an NYHA measure obtained before 3 months after the procedure. This methodological decision affected 15 patients, with the consequence that an ordinal logistic regression model could not be performed because there was only 1 patient in the SM group with NYHA III. Table 3 is a contingency  table describing the evolution of NYHA functional class pre- and post-procedure, with a minimum of 3 months after the procedure was carried out.

**Table 3:** Pre- and post-procedural New York Heart Association functional class

NYHA pre	NYHA post			
ASA	I	II	III	Total
II	1 (20%)	2 (40%)	2 (40%)	5
III	2 (11%)	7 (39%)	9 (50%)	18
Total	3	9	11	23
SM	I	II	III	Total
II	10 (100%)	0 (0%)	0 (0%)	10
III	32 (76%)	9 (21%)	1 (2%)	42
IV	3 (75%)	1 (25%)	0 (0%)	4
Total	45	10	1	56

ASA: alcohol septal ablation; NYHA: New York Heart Association; SM: septal myectomy.

**Figure 1:**
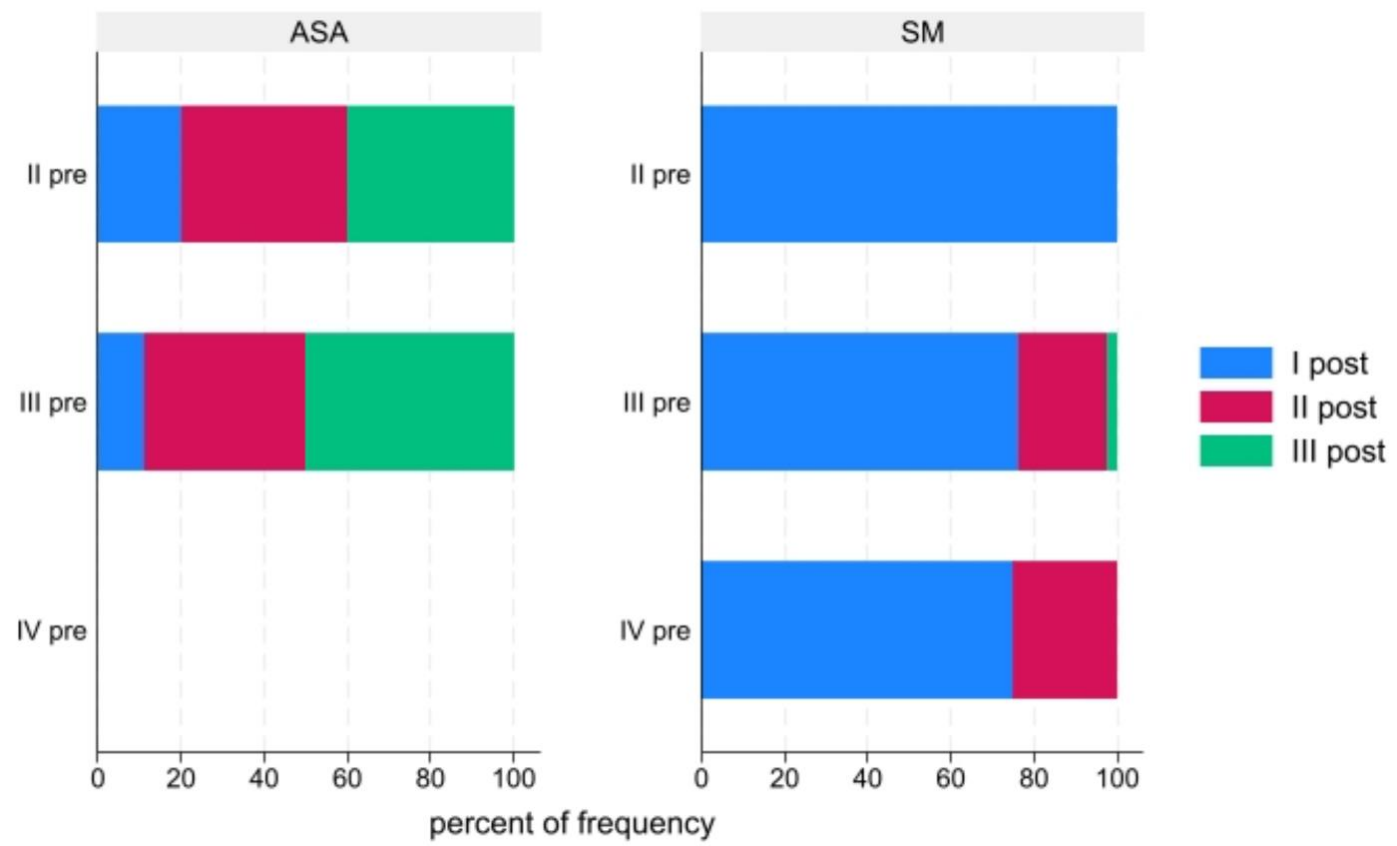
New York Heart Association group percentage pre- and post-alcohol septal ablation/septal myectomy. The colour boxes indicate the post-procedure New York Heart Association percentage according to their corresponding previous New York Heart Association values (horizontal boxes).

Post-ASA patients had a probability of being in NYHA functional classes as follows: I, 13%; II, 39%; and III, 47% respectively; post-SM patients, I, 80%: II, 17.8%; and III, 1.78%, respectively. A total of 2/18 (11%) patients who were in III pre-ASA achieved NYHA I after the procedure; meanwhile, in the SM group, 32/42 (76%) of patients in NYHA III achieved NYHA I after the procedure (Fig. 1). No difference in angina (12.6% ASA vs 2.5% SM; *P* = 0.115) or in syncope (0% vs 0%) was observed, but dyspnoea presented with a higher prevalence in the ASA group (75% vs 10.3%, *P* < 0.001). As expected, most patients in both groups continued with beta-blocker therapy, but the therapy could be reduced to a greater extent after SM (preprocedure ASA 100% vs SM 94.9%; post-procedural ASA 96% vs SM 71.8%; *P* = 0.034). Meanwhile, patients in the SM group could discontinue most of the CCB and disopyramide (preprocedure CCB 48% vs 15.4%; post-procedural 16% vs 3.8%; *P* = 0.054, preprocedure disopyramide 20% vs 59%; post-procedural 36% vs 1.3%; *P* < 0.001).

**Figure 2:**
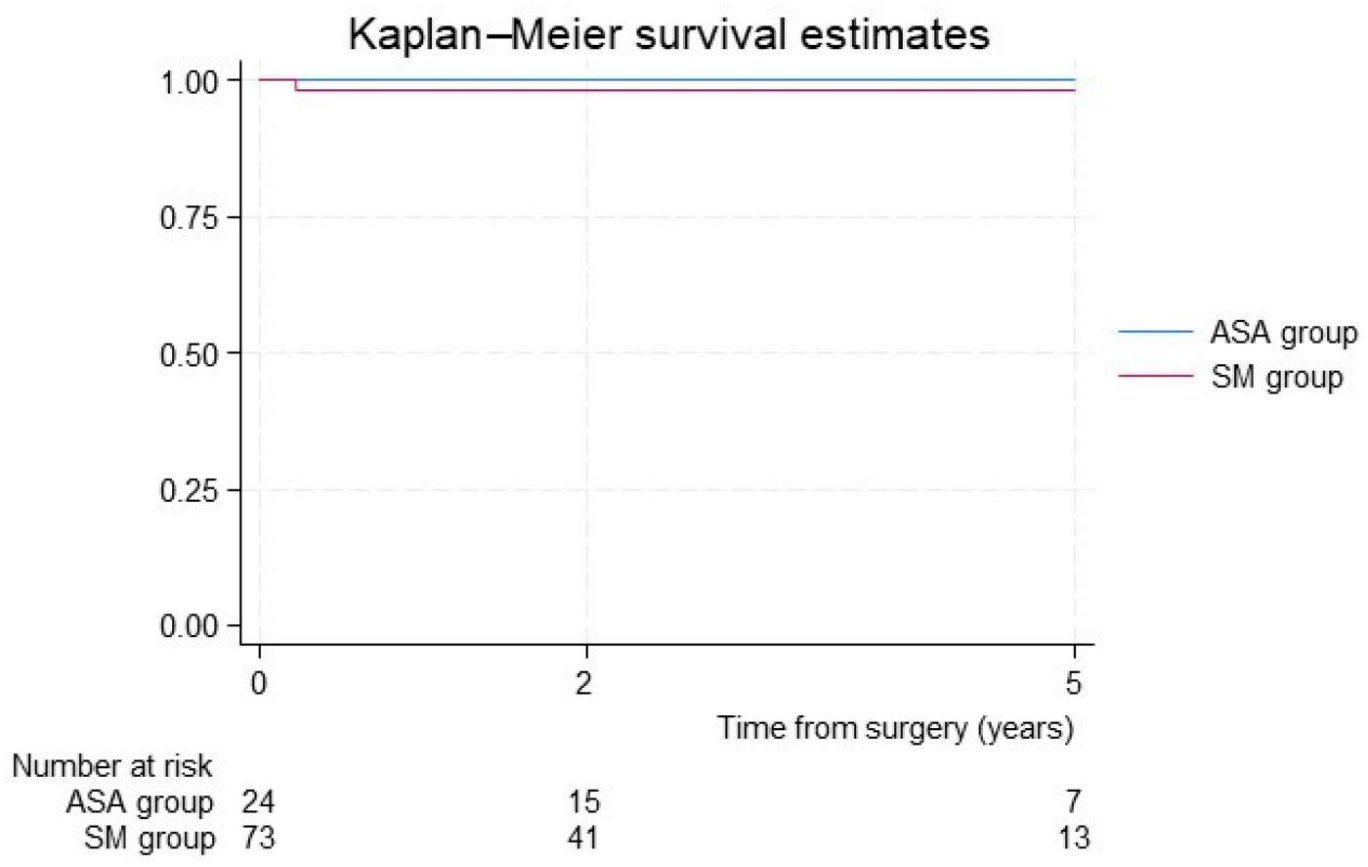
Kaplan–Meier survival functions.

The requirement for a second procedure was higher in the ASA group (11.53% vs 1.28%; *P* = 0.006): 2 patients required an SM and one patient, a redo-ASA procedure. Meanwhile in the SM group, 1 patient required an ASA due to persistently high gradients. In-hospital mortality was similar in both groups (*n* = 0 in ASA and *n* = 1 in SM). This patient’s death was due to respiratory causes on a comorbid patient. Survival follow-up was similar between both groups, as shown in Fig. 2 with a Kaplan–Meier analysis.

## DISCUSSION

Our main finding is that SM reduces basal and Valsalva gradients to a greater extent in comparison to ASA. In accordance with our results, other studies and meta-analyses have shown a higher decrease in the gradients after SM [14–16, 18]. SM also achieves a better NYHA class and reduces the need for medical treatment in comparison to ASA. As reported by Maron *et al.*, SM improves symptoms in >90% of patients by ≥ 1 NYHA functional class, 75% of them becoming asymptomatic [19]. These results are comparable to those seen in our series, with 81.16% patients achieving NYHA I after SM, 77% of whom were previously NYHA III.

Inadequate septal excision at the initial procedure leads to recurrent symptoms and LVOT gradients [14]. Regrettably, measurement of LVOT gradients with TEE may be challenging due to difficulties in aligning the Doppler beam [20]. Therefore, the use and combination of intraoperative TEE and direct measurement of gradients are crucial to ensuring the adequacy of the SM. Regardless of our small volume, which is in contrast to that at other high-volume centres, our results are comparable to those of larger series. This important finding highlights that in centres with an active HOCM program and with a strict action protocol focused on reducing gradients in LVOT, similar results can be obtained. Ashikhmina *et al.* confirmed years ago the importance of measuring intraoperative LVOT gradients before performing an SM due to the variations in these gradients in patients under general anaesthesia. The measurement of gradients preoperatively may help the surgeon performing the SM to interpret residual gradients after the procedure. We used 2 provocative manoeuvres: provocation of a premature ventricular contraction (Brockenbrough-Braunwald-Morrow manoeuvre) and dobutamine infusion. Gradients with provocative manoeuvres greater than 15 mmHg were considered suboptimal, and they were interpreted as being due to incomplete resection [21]. In these cases (20% of our series), a new clamping was performed to extend the myectomy further. In other series, as in the Mayo Clinic, this number was as low as 4% [21], probably due to their higher confidence with the initial resection. In contrast, after the ASA procedure, immediate gradient reduction is not achieved in all patients. The decrease in gradients starts as soon as 2 days post-ASA, but it is more evident 3 months after the procedure, with resolution of the oedema of the infarcted area [22–23]. The difference in being extremely strict with the basal and induced gradients could explain the better results achieved with an operation.

Some series have reported that SM solves >95% of MR and SAM [14]. Numerous aspects of the mitral valve take part in MR, such as hypertrophied and mispositioned papillary muscles, anterior leaflet enlargement or anomalous chordae tendinae. As recommended in other large series, the mitral valve should be thought of as a component that worsens LVOT obstruction and should be treated if necessary [16, 17, 24, 25]. In our series, mitral valve repair with vertical plication was noted in 21.79% of the patients, and resection of the anomalous chordae tendineae was carried out in 16.67%. The higher percentage of repaired mitral valves in comparison to previous series may be related to a different HOCM phenotype, and it emphasizes the advantage of performing a surgical treatment to correct the associated mitral pathology. We performed vertical plication in those with an elongated anterior mitral valve leaflet, with a length >34 mm, as recommended by other authors [26].

In our series, one patient in the SM group presented a transient deficit without computer tomography expression, probably in relation with some muscle debris migration. The need of a PM implant and the requirement for a second procedure were higher in the ASA group, comparable to previous reports [1, 4, 15, 16, 27, 28]. ICU and hospital LOS were comparable to those previously reported [16, 18, 27, 28]. Further, survival after SM and ASA is similar to survival among an age- and sex-matched population [8, 14, 27]. On the other hand, in some series, survival after SM was better than that of an age-, sex- and race-matched population [16]. Also, with the use of novel medical treatment, the patients referred for interventional options are expected to be elderly and with greater comorbidities [28].

Regarding worldwide invasive management of patients with HOCM, the 2014 European Society of Cardiology Hypertrophic Cardiomyopathy (HCM) Guideline does not state a clear attitude in favour of either procedure [29]. Conversely, the 2020 American Heart Association guidelines for the diagnosis and treatment of HCM state that myectomy should be preferred rather than ASA [30]. Additionally, the latter recommend ASA when it is performed in experienced centres in patients for whom surgery is contraindicated or considered unacceptably high risk because of serious comorbidities or advanced age [30].

Moreover, close follow-up of patients before and after each procedure in our Inherited Heart Disease Unit resulted in a precise adherence to treatment and lifestyle changes that benefit all patients. As endorsed in the 2020 AHA Guidelines for Diagnosis and Treatment of Patients with Hypertrophic Cardiomyopathy with a class 2a recommendation, multidisciplinary HCM centres with expertise are important to enhance care for patients with HOCM [29]. The main goal of these centres is to enhance the care and counseling of patients and their families as well as to provide diagnoses, treatment evaluations and thorough follow-ups [29]. Furthermore, the 2023 ESC guidelines for the management of cardiomyopathies validate with a class Ic the importance of a multidisciplinary team with expertise in the diagnosis and management of cardiomyopathies [30]. We have such a team in our Inherited Heart Disease Unit, comprised of cardiologists, cardiac surgeons and an interventional cardiologist, that provides medical guidance and options for invasive strategies. For this, among other reasons, our results may be superposed to the series of high-expertise centres.

The results of this study might impact the choice of procedure in individual patients. Furthermore, we encourage other high-volume centres to perform an international multicentre randomized trial. When medical therapies fail, SM should continue as the gold standard intervention for HOCM, even in in low-volume centres, whereas ASA remains an option for those very high risk and comorbid patients.

### Limitations

This study has several limitations. First, it is a single-centre study in which the SM and ASA procedures are performed by only 1 surgeon/interventional cardiologist. Second, most of the patients were referred for surgery, which is an important bias selection. Third, during the ASA procedure, no post-procedural gradients are measured in the cardiac catheterization laboratory; they are examined only 3 months after the ASA procedure. Moreover, after performing an inverse-probability weighted regression-adjustment analysis, we determined that the groups may not be comparable; thus we had to perform a traditional multivariable analysis. There are no other statistical methods that could ensure a correct comparison between these 2 groups. Furthermore, due to the small sample, our study could be underpowered to detect other clinically relevant differences. Finally, some patients are followed in their reference hospitals and not in our Inherited Heart Disease Unit, which would account for follow-up loss and variations in medical management.

## Data Availability

The data that support the findings of this study are available on request to the corresponding author. To preserve the privacy of the participants included in this study, the data are not openly available

## Notes

Presented at the 37th Annual Meeting of the European Association for Cardio-Thoracic Surgery, 4–7 October 2023, Vienna, Austria.

## ABBREVIATIONS AND ACRONYMS

ASA: Alcohol septal ablation
CCB: Calcium channel blocker
CPB: Cardiopulmonary bypass
EuroSCORE II: European System for Cardiac Operative Risk Evaluation II
HCM: Hypertrophic cardiomyopathy
HOCM: Hypertrophic obstructive cardiomyopathy
LOS: Length of stay
LVOT: Left ventricle outflow tract
MR: Mitral regurgitation
NYHA: New York Heart Association
OR: Odds ratio
SAM: Systolic anterior motion
SM: Septal myectomy
TEE: Transoesophageal echocardiography
